# Implementation of an interactive virtual microscope laboratory system in teaching oral histopathology

**DOI:** 10.1038/s41598-022-09473-6

**Published:** 2022-03-31

**Authors:** Jia Qing, Gu Cheng, Xiao-Qi Ni, Yi Yang, Wei Zhang, Zhi Li

**Affiliations:** 1 The State Key Laboratory Breeding Base of Basic Science of Stomatology (Hubei-MOST) and the Key Laboratory of Oral Biomedicine Ministry of Education, School and Hospital of Stomatology, Wuhan University, 237 Luoyu Road, Wuhan, 430079 China; 2 School of Stomatology, Peking University, Beijing, China; 3 Department of Endodontic, School and Hospital of Stomatology, Wuhan University, Wuhan, China; 4 Department of Oral and Maxillofacial Surgery, School and Hospital of Stomatology, Wuhan University, Wuhan, China

**Keywords:** Medical research, Oral diseases

## Abstract

Laboratory course acts as a key component of histopathology education. Recent trends of incorporating visual and interactive technology in active and inquiry-based learning pedagogical methods have led to significant improvement of histopathology laboratory courses. The present work aimed to describe interactive virtual microscope laboratory system (IVMLS) as a virtual platform for teaching histopathology in order to improve the quality and efficiency of teaching. The system is based on interactive technology and consists of interactive software, slide-reading software, teaching resources and integrated auxiliary equipment. It allows real-time interaction between teachers and students and provides students with a wealth of learning and review materials. In order to evaluate the effectiveness of the system, we conducted a comparative study with the use of light microscope (LM) as a method. Specifically, we compared the results of six assignments and one laboratory final exam between IVMLS group and LM group to analyse the impact of IVMLS on students' academic performance. A questionnaire survey was also conducted to obtain students' attitudes and views on this system. There was no overall difference in assignment performance between IVMLS group and LM group. But laboratory final test grades increased from a mean of 62% (43.8–80.0, 95% CI) before to 83% (71.0–94.2, 95% CI) after implement IVMLS, suggesting highly significant (p < 0.001) improvement on students' histopathology laboratory performance. Feedback of the questionnaire was positive, indicating that students were satisfied with the system, which they believed improved student communication and teacher-student interaction, increased learning resources, increased their focus on learning, and facilitated their independent thinking process. This study proves that IVMLS is an efficient and feasible teaching technology and improves students' academic performance.

## Introduction

With the development of the Internet, the concept of e-learning is introduced. In essence, e-learning is the use of Internet technology to provide learners with available information or knowledge, without time or geographical constraints to enhance knowledge and performance^1^, and it is becoming a new paradigm of education^2,3^. Historically, there have been two common e-learning models: distance learning and computer-assisted teaching. The latter uses computers to help provide independent learning and teaching multimedia packages^1^. In fact, computer-assisted instruction has been widely used in higher education in the past 20 years. There are abundant researches on the influence of technology on teaching. It is found that the mode of presenting learning content will significantly affect the learning process and thus the learning effect^4^. When the learning process is integrated with multimedia tools, students are more interested in the learning topic^5^. It’s found that the design of the relevant learning application will provide an efficient learning method and use it to improve students' scores ^6,7^. Evidence also supports that teaching with integrated technical tools can not only help teachers improve teaching quality, but also help students improve their learning level^8^. In recent years, computer simulation^9^ and Web3D technologies^10^ and other interesting and practical technologies have been applied in teaching practice and brought changes to classroom forms. Histopathology is no exception.

Histopathology is an essential part of undergraduate dental education. However, due to the complexity of histological structure and the difficulty of integrate histological structures with physiological functions, pathology is often considered as one of the most difficult courses for students^11^. As a detail-oriented biomedical course that uses vision to reveal the truth^12,13^, laboratory course is induced as a key component of histopathology education. It aims to develop students' ability to recognize subtle morphological differences in tissue characteristics and integrate their knowledge of organ function with observation of two-dimensional microscopic images^14–16^. This is a complex, multi-step process, usually facilitated by interaction between learners and teachers^17^.

Direct interaction between students and teachers during laboratory sessions is identified as immediate informal feedback in the formative assessment^17^, which is defined as teaching tools generating feedback information that benefits students during the learning process and leads to enhanced learning outcomes^18^. In laboratory sessions, tests, lab reports, classroom tasks, teacher–student interactions are all included in this category. Teachers use these tools to gain insight into misinterpretations and gaps in students' understanding and to provide constructive feedback to students^17^. The opportunity to provide a formative assessment has been identified as a significant benefit to student learning^18^. However, informal feedback may never reach shy students who do not like to ask questions or seek clarification^19^, leading to marginalization and failure to reach their full potential. Improving university instruction to increase engagement and retention of underrepresented students is one of the things that many teaching participants have been trying to achieve^20,21^.

In laboratory sessions, students are instructed to study tissue structures outlined in the previous lectures in detail through a light microscope or VM. They need to understand the characteristics of each tissue types and use these characteristics to distinguish them. In addition, students are also required to understand the basic unity of form and function at the micro level. However, students often encounter difficulties in laboratory sessions due to the lack of microscopic experience, the complexity of histologic images and the difficulty of integrating static histological images with dynamic physiological functions^22^. Recent trends of incorporating visual and interactive technology in a variety of active and inquiry-based learning pedagogical methods^11,23,24^ have led to significant improvement of oral histopathology laboratory courses.

In terms of interaction, many technologies have been gradually employed and achieved good results^17,25^. For example, interactive whiteboards proposed by Jain ^26^ can be used in laboratory courses to create an interactive learning environment^11^. As for virtual technology, since R Ferreira put forward the concept of virtual microscope (VM) in the late 1990s^27^, this technique has been implemented in a variety of educational venues^28^, such as cytology^29–32^, hematology^33^ and dermatopathology^34,35^. In order to meet the teaching needs of various disciplines, VM has developed from standard virtual slides to 3-dimensional (3-D) slides^30,36–38^ in the past 30 years. Previous reports have described that the advantages of VM include increased accessibility, ease of use and perfect emulation of traditional microscope^28,39–42^. Given these advantages, medical colleges worldwide around the world are increasingly incorporating the VM platform into histopathology laboratories to improve students' learning experience in microscope laboratory sessions^42–44^.

IVMLS is a system that perfectly incorporates both visual and interactive technology. It is designed to promote the interaction between teachers and students, thus providing an ideal virtual platform for teaching and learning. IVMLS is often composed of interactive software, teaching resource, slide-reading software and integrated auxiliary equipment. Virtual slides are one part of teaching resource and incorporated into this system. The biggest feature of IVMLS is to connect the computers of teachers and students in the laboratory, so as to improve the teaching efficiency^45^. IVMLS can be used to control students’ computers in the laboratory, monitor the tabs and programs displayed on their screen and provide a platform for teachers and students to interact and share resources. The purpose of this study was to investigate the teaching effect of IVMLS and evaluate students’ attitude towards this technology. The impact of this teaching method is based on results of questionnaire survey and students' performance in the homework assignments and laboratory tests.

## Materials and methods

### Course context

Oral histopathology is an upper-level undergraduate course for dental students of grade four. It is an important subject in basic oral medicine and a bridge between basic oral medicine and clinical oral medicine. For medical students, learning this course is of great significance for correctly understanding the essence of oral diseases and learning to correctly diagnose and treat diseases. And laboratory courses play as a key component of histopathology education. 16 h were assigned for lectures and 18 h for laboratory. The course consists of 8 lectures, each lasting two hours, followed by six 3-h laboratory sections.

The study was performed during the laboratory sessions of the Oral Histopathology course. This course was given in 2018 and 2019. Specifically, the students admitted in 2015 took this course in 2018, while the students admitted in 2016 took the course in 2019. Before laboratory sessions, extra time was arranged to train the students with basic skills and precautions for using a LM or the slide-reading software NDP. View 2. In order to achieve better teaching results, laboratory courses were arranged to closely follow after each lecture to consolidate theoretical knowledge. There are also cases where a laboratory course is carried out after two consecutive lectures. Therefore, there are eight lectures and six laboratory courses instead of six lectures and six laboratory courses. However, it can be guaranteed that the tissue types studied in each laboratory session had been introduced in the previous lectures.

### Participants

All the undergraduates, 156 in total, who was admitted in our school of stomatology in 2015 and 2016 participated in this study. 77 students (25 males, 52 female) who entered university in 2015 were taught by traditional teaching method in 2018. 79 students (32 males, 47 female) who entered university in 2016 accepted the proposed teaching method of IVMLS in 2019. All the participants had passed the entrance criteria of the University. The enrolment standards along with teaching plans of the two sessions remained unchanged in these two years. Therefore, the academic level of the two groups was principally consistent. Students in the two groups were of similar age (20–22 years old, p > 0.05) when they participated in this study. In addition, there was no significant difference (p > 0.05) between the two groups in male–female ratio (Table 1). Thus, anthropic characteristics of two groups were comparable in this study. All the participants had previously accepted traditional laboratory teaching method and knew the basic knowledge of using microscope. Institutional review board (IRB) approval of School and Hospital of Stomatology, Wuhan University was obtained for this study, and all study protocols were approved. All research was performed in accordance with relevant guidelines. All participants involved in this study gave their informed consent.

**Table 1:** Descriptive data of participants enrolled in this study.

Demographics	Total	LM group	IVMLS group	*p*-value
Students; n	154	77	79	
Age; years (± SD)	21.0 (± 0.8)	21.1 (± 0.8)	21.0 (± 0.8)	0.531
**Sex; n (%)**				
Female	99 (63.5%)	52 (67.5%)	47 (59.5%)	0.297
Male	57 (36.5%)	25 (32.5%)	32 (40.5%)	

*LM* light microscope, *IVMLS* interactive virtual microscope laboratory system, *n* number of students.

### Laboratories

#### Traditional histopathology laboratory for LM group

The traditional way of teaching was implemented in the traditional histopathology laboratory in 2018. This laboratory consists of the following components: (1) a computer for the teacher and the connected projector; (2) a LM for the teacher; (3) several LMs for students; (4) glass slides that were carefully selected to ensure high quality and definition; and (5) other auxiliary equipment.

#### Advanced histopathology laboratory for IVMLS group

**Figure 1:**
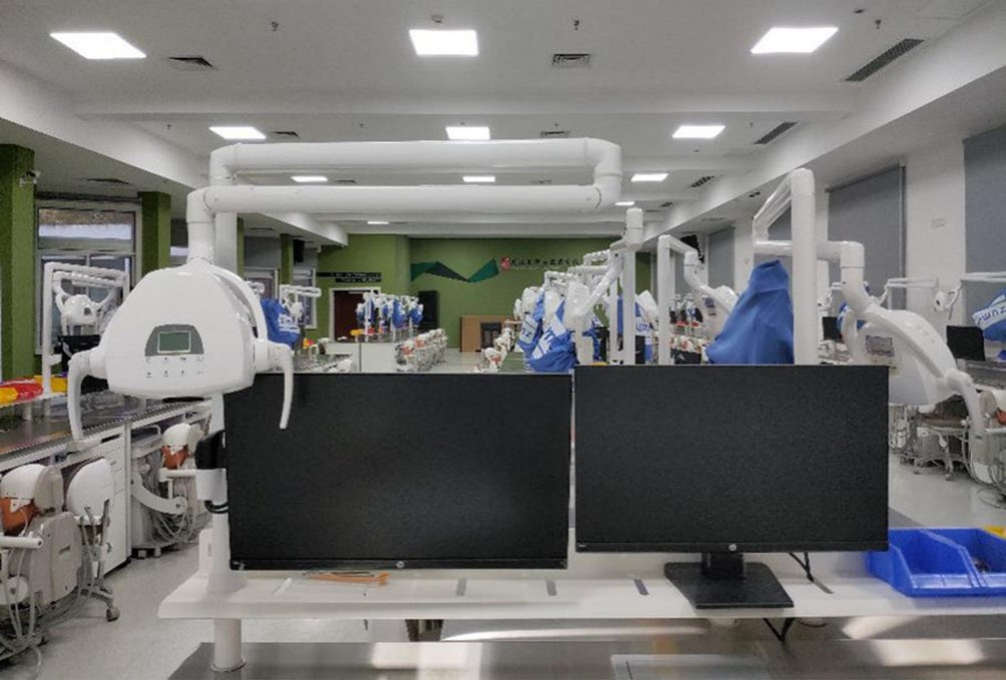
Advanced histopathology laboratory is composed of a computer for the teacher, several computers for students, interactive virtual microscope laboratory system (IVMLS) and other auxiliary equipment.

Students in Grade 2016 accepted the proposed teaching method of IVMLS in the advanced histopathology laboratory in 2019. This laboratory consists of the following components: (1) a computer for the teacher; (2) several computers for students; (3) IVMLS; and (4) other auxiliary equipment (Fig. 1).

**Figure 2:**
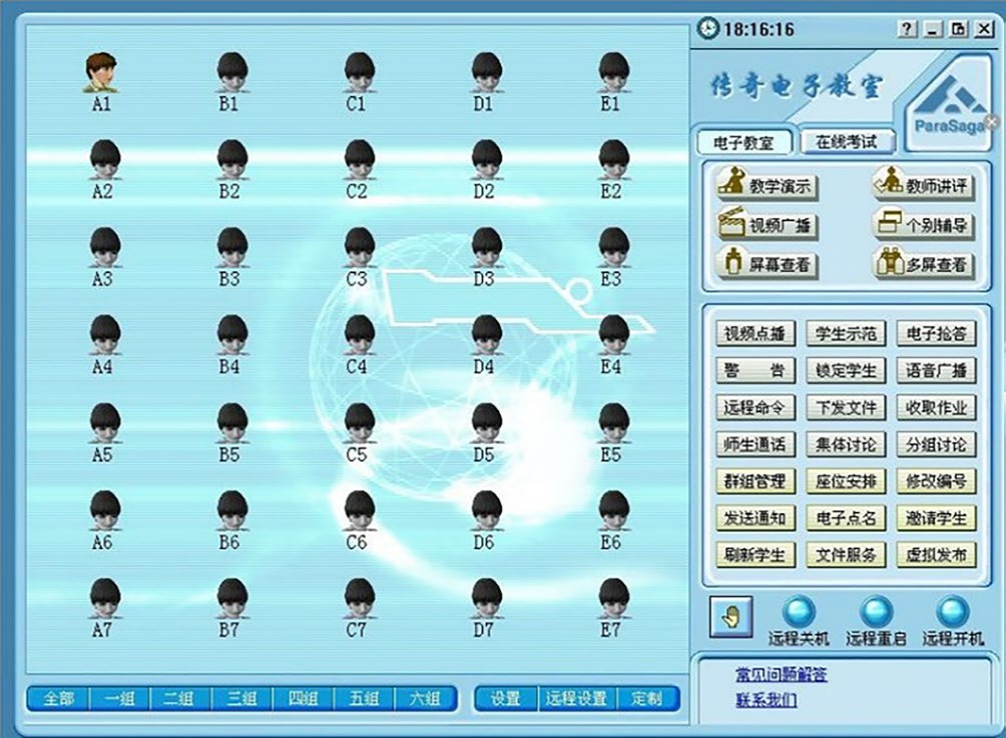
Teachers can share teaching resources, interact with students, share their computer interface with each student's computer in real time, and monitor the tabs and programs displayed on their screen via ParaSaga EClass.

**Figure 3:**
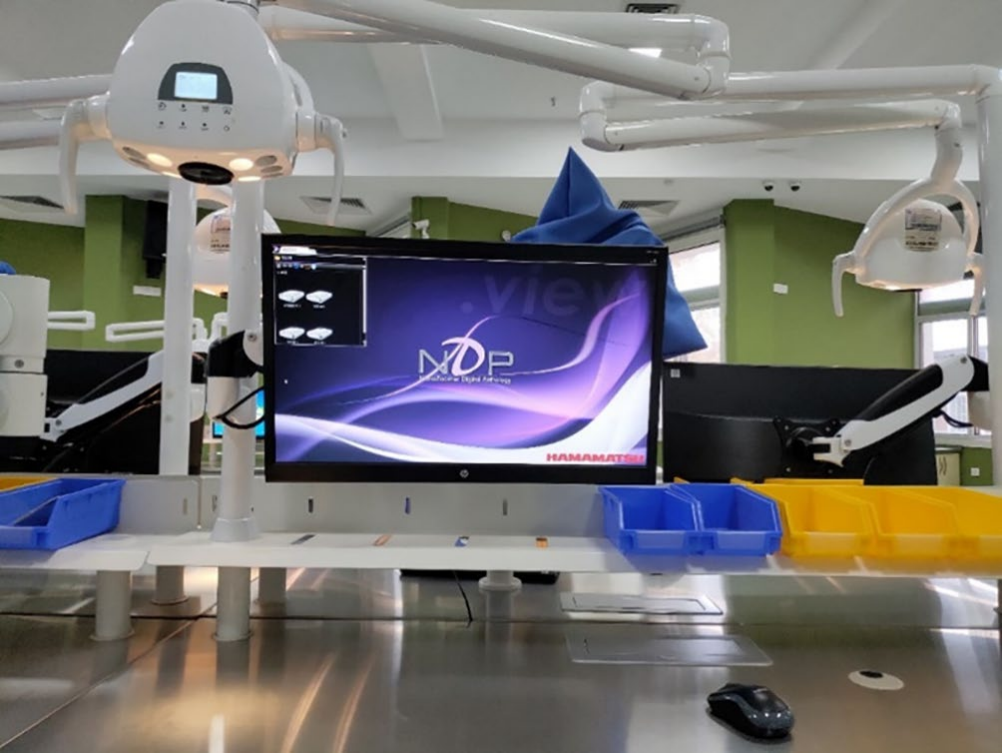
The homepage of NDP. View 2. Virtual slides can be selected and viewed by clicking on the folders in the upper left corner.

**Figure 4:**
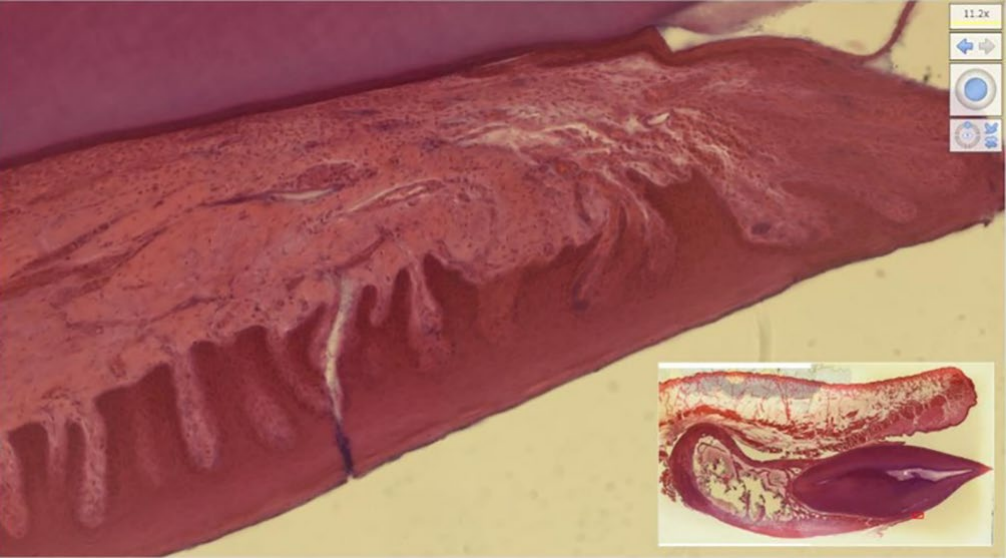
NDP. View2 allows the digital slide images to be dragged and clicked with the mouse to simulate the scanning of the corresponding glass slides under a light microscope.

IVMLS often comprises interactive software, teaching resource, slide-reading software and integrated auxiliary equipment. The interactive software we employed in this study is ParaSaga EClass (Lenovo, Beijing, China), via which teachers can share teaching resources, interact with students, share their computer interface with each student's computer in real time, and monitor the tabs and programs displayed on their screen (Fig. 2). Students can also interact with their teachers proactively in this platform. What’s more, they can receive and store the digital teaching resources shared by their teachers, cut and paste a region within a slide to produce a .jpg or .tiff file that can be downloaded into their USB flash disk and submit their assignments and papers online, if assigned. Teaching resources include teaching plans, virtual slides and PowerPoint documents which delivered the relevant theoretical knowledge. The virtual slides are 3-D slides and come from the teaching virtual slide database of China from Peking University. The slide-reading software is named NanoZoomer Digital Pathology. View 2 (NDP. View 2, Hamamatsu photonics K.K, Japan) (Fig. 3), with which the virtual slide images can be dragged and clicked with the mouse to simulate the scanning of the corresponding glass slides under an LM (Fig. 4).

### Conduct of laboratory courses

#### Traditional laboratory courses

Traditional laboratory courses started with a presentation describing the relevant theoretical knowledge via PowerPoint documents. Then, students observed the slides by themselves and discussed with others. In this stage, students needed to complete some tasks, such as finding the landmark structure of a certain pathological tissue. If they had any questions, they could raise their hands or go to the teacher to ask questions. When teachers observed typical tissue structures under their own microscopes, students can also go to the teacher's microscope one by one to observe them. At the end of the class, the teacher answered the frequently asked questions and raised new questions. Students can get access to the glass slides and microscopes after class as long as they apply to the teacher.

#### Advanced histopathology laboratory courses

For IVMLS group, at the beginning of the course, teachers shared their own computer interface to students in real time for PowerPoint presentation of theoretical knowledge, so that the content of the document is clearly visible to everyone. Then, students were instructed to explore the slides by themselves, with the same tasks as the previous group. But they could not talk to each other. Because the purpose of this stage was to guide students to think independently, to reason and to deepen their recognition of organization types and basic concepts^46–48^. With the slide-reading software, students were allowed to take screenshots of important structures and mark them with arrows, circles, or other patterns. If they had any question, they could send messages to their teachers using the ‘Hand Up’ button in the system, without disturbing others. At the next stage, teachers shared their computer interface again and used cursor or annotation mode to accurately indicate the specific characteristics of tissue types to students. Teachers could also use this period of time to answer the questions asked frequently and assign homework. Lastly, students should observe the slides again and discuss freely with their classmates. For review purposes, the slide-reading software, along with all the slides and screenshots, could be freely downloaded in students’ own USB flash drives. Throughout the whole teaching process, teachers could easily supervise the students by monitoring their computer interface. They could also utilize a black screen and text warnings to remind students who were not studying attentively.

### Assessments and assignments

Assessments of two types were used: homework assignments and laboratory final test. There were six homework assignments, which accounted for 30% of the final grade. The final examination represented the remaining 70% of the final grade. Homework was arranged before the free discussion part, and students were required to draw the designated tissue structure after class. In the laboratory final test, students were required to identify tissue structures in the pictures. There were 2–5 structures indicated by arrows in each picture, and 40 in total. These pictures were made into a PowerPoint document, and the switching time of each image was controlled to one minute by PowerPoint software. Students in LM group took the examination in 2018. And the other group took the examination in the same classroom in 2019. The PowerPoint document were identical for two groups. Teachers and textbooks remain unchanged in these 2 years.

Statistical analysis was performed using IBM SPSS Statistics for Windows Version 24.0 (IBM Corp., Armonk, NY, USA). Statistical analysis involving both groups was performed using the *t*-test of students for paired data, wherein statistical significance was accepted at the level of *p* < 0.05.

### Questionnaire survey

The questionnaire was designed on the basis of previous researches^40^ to obtain information about the students' perception of IVMLS. These surveys were sent to students utilizing a web-based survey tool. As students had previously accepted the traditional teaching method, their opinions could provide some clues to the educational value of this shift from traditional teaching method to IVMLS. The survey assessed student satisfaction and the impact of IVMLS on factors that affect learning effectiveness, including student discussion, teacher-student interaction, independent thinking, focus on learning, learning resources and ease of tools. The survey had questions that used the five-point Likert type scale (1 = Strongly disagree; 2 = Disagree; 3 = Neutral; 4 = Agree; 5 = Strongly agree; 1 = poor rating and 5 = excellent rating) and open-ended questions. All mean values provided are accompanied by respective standard deviation (± SD). correlation coefficient (r), Kendall's tau B and Cronbach's alpha statistical tests were performed to assess correlation and reliability using IBM SPSS Statistics for Windows Version 24.0 (IBM Corp., Armonk, NY, USA). It was calculated that the Cronbach's alpha of the questionnaire was 0.971, the correlation coefficient of the questionnaire was greater than 0.6, and the Kendall's tau p value p(τ) was less than 0.001.

## Results

Students in the two groups were of similar age (20–22 years old, p = 0.914) when they participated in this study and there was no significant difference (p = 0.685) between the two groups in male–female ratio. Table 2 shows a comparison of students' assignment and laboratory final test scores in the histopathology laboratory sessions. The average scores of each assignment of the two groups fluctuated around 80%. Results showed that there was no significant difference in the overall performance of homework assignments between the two groups of students (p = 0.235). Performance of LM group was significantly better than that of IVMLS group in the second (p = 0.030), third (p = 0.004) and fifth homework (p < 0.001), but there was no significant difference between the two groups in the first (p = 0.358) and sixth homework (p = 0.072). Students in IVMLS group only performed better than students in LM group in the fourth assignment (p = 0.007) (Table 2).

**Table 2:** Comparison of student performance in homework assignments and laboratory final test before and after the application of IVMLS.

Assignments	LM group (n = 77) %Mean (± SD)	IVMLS group (n = 79) %Mean (± SD)	*p*-value
Assignment 1	81.6 (± 11.2)	79.7 (± 13.2)	0.358
Assignment 2	82.8 (± 6.1)	80.7 (± 6.1)	0.030
Assignment 3	83.4 (± 6.6)	80.6 (± 5.4)	0.004
Assignment 4	79.4 (± 7.1)	82.5 (± 7.2)	0.007
Assignment 5	87.1 (± 4.1)	83.7 (± 5.9)	0.000
Assignment 6	86.5 (± 4.5)	87.9 (± 5.1)	0.072
Total assignments	83.4 (± 4.9)	82.5 (± 4.9)	0.235
Laboratory final test	61.9 (± 18.3)	82.6 (± 11.6)	0.000

*LM* light microscope, *IVMLS* interactive virtual microscope laboratory system, *n* number of students.

Two students in LM group did not take the final test, so only 75 test scores were received from LM group. For IVMLS group, all 79 students took the laboratory final test. Histopathology grades ranged between 0 and 100% with a class average percentage grade of 62 (95% CI of 43.6–80.2) before the integration of IVMLS. As for students in the other group, their grades increased to a range between 42 and 99% with a class average percentage of 82.6 (95% CI of 81.0–94.2). Comparison of student performances on the laboratory final test before and after the implementation of IVMLS showed a significant improvement in the grades of students (p < 0.001) (Table 2).

**Figure 5:**
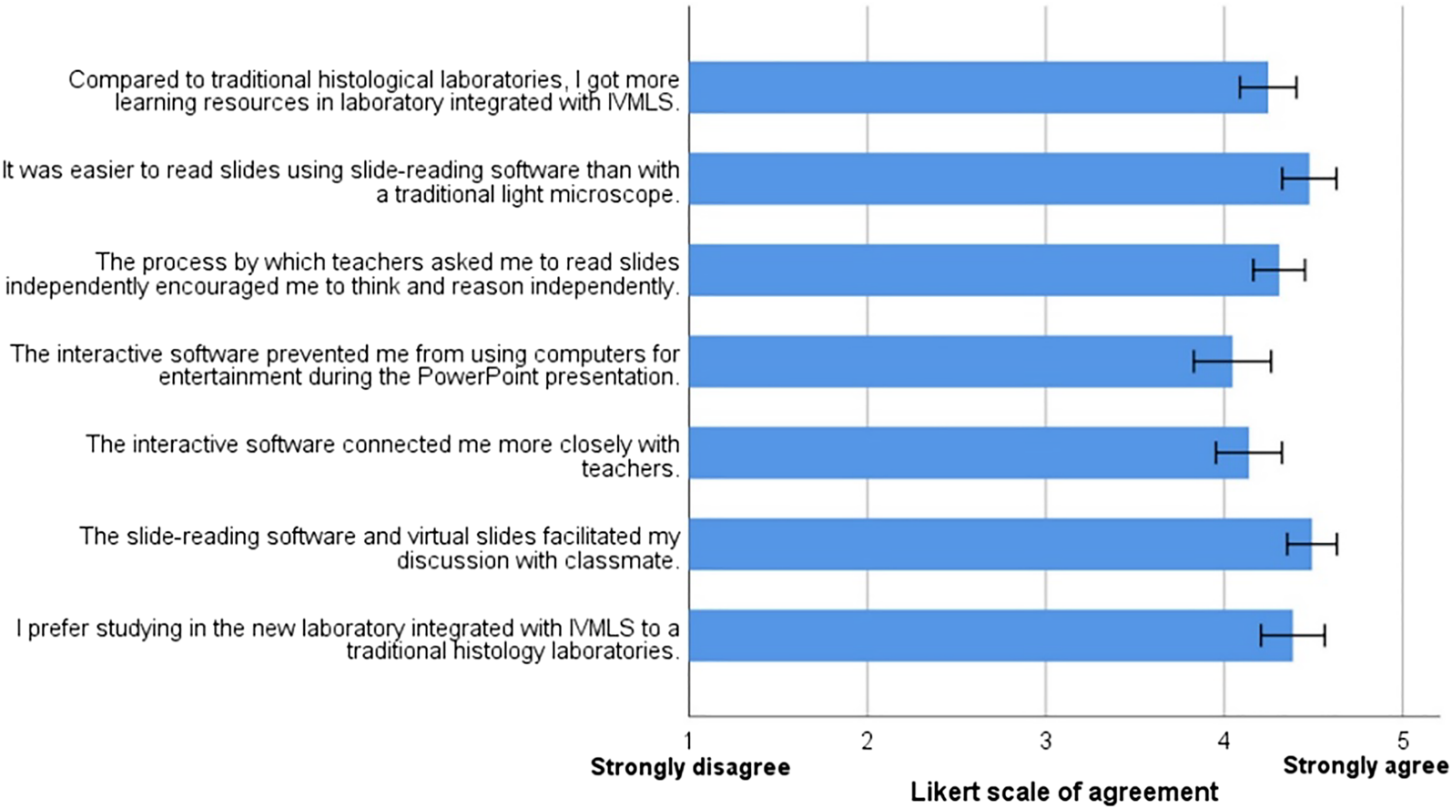
Perceptions of students toward interactive virtual microscope laboratory system (IVMLS). A horizontal bar graph shows means ± standard deviation of agreements expressed on the Likert scale (1 = strongly disagree, 5 = strongly agree).

Students’ perceptions toward IVMLS were shown in Fig. 5. The questionnaire results were positive. Recovery rate of the questionnaire survey reached 82.3%. The questionnaire results indicated that the students were satisfied with the proposed teaching method and it has achieved positive effects. Most students agreed that they prefer studying in the new lab integrated with IVMLS to a traditional histopathology lab (89.23%). As for the impact of IVMLS on factors that affect learning effectiveness, almost all the students (96.93%) held the view that the slide-reading software and virtual slides included in IVMLS facilitated their communication and discussion with their classmates. The majority of the participants felt that the interactive software connected them more closely with their teachers (81.54%) and prevented them from using computers to do things that have nothing to do with learning during the PowerPoint presentation (76.93%). The vast majority of students believe that in the reformed histopathology laboratory sessions, the process by which teachers asked them to read slides independently encouraged them to think and reason independently (93.85%). The same degree of agreement was reached on the ease of tools, with 93.85% of students saying it was easier to read slides using slide-reading software than with a traditional light microscope. Regarding learning resources, most students agreed that they got more learning resources in IVMLS (89.23%).

In addition, students were given the opportunity to make written comments at the end of the survey. The responses were supportive of the proposed teaching method of IVMLS and provided some reasons why students preferred the proposed teaching mode, including the phrases ‘better visualization’, ‘time-saving’, ‘more accessible’, ‘ease of use’ and ‘fun’. Students also raised some problems regarding the stability of the IVMLS. Specifically, students pointed out issues such as the occasional freezing of the shared interface and the possibility of losing control within a small proportion of computers whilst in the middle of the class. Students further suggested that the stability of the IVMLS should be strengthened, and the fluency of the network should be improved. Students also gave some other valuable suggestions, such as combining traditional light microscopes with IVMLS, providing an example of specific structures for each slide, and improve the microphone and signal transmission system.

## Discussion

One of the key points of histopathology learning is to identify diagnostic-related areas from the entire sample and identify the tissues and cells in them. Understanding the complexity of tissue organization, function and pathological processes^49,50^ through the memorization of superficial, irrelevant information is a continuous struggle for students^11,51^. The rapid progress of science and technology provides a platform for the development of laboratory sessions of oral histopathology^40^. The introduction of interactive whiteboards^11,26^ and other technologies provides a new platform for interaction between teachers and students^17,25^, while VM makes the teaching platform transfer from offline to online. IVMLS described here integrates both interactive technology and visual microscopy and puts emphasize on teacher-student interaction, inquiry-based learning and reasoning^24,46,47,52,53^.

### A flip of classroom mode

By comparing students’ laboratory results before and after IVMLS implementation, it is found that IVMLS implementation was associated with improved laboratory test scores and, concurrently, a decreasing failure rate and an increasing high score rate, thus narrowing the score distribution. This may be the result of a better learning environment supported by IVMLS. The characteristics of today's Millennial students necessitate instructional strategies that provide clear structure, support group learning, actively engages students in the learning process, and provides feedback to students to monitor their progress^54–56^. Students’ feedback of their experience of using IVMLS was very favourable. They agreed that the system facilitated histopathology learning by enhancing classroom discussion, facilitating teacher-student interaction, promoting thinking and reasoning, and helping to increase learning focus.

Interaction between teachers and students are seen as an important factor influencing teaching results^53,57^. The interactive software described here allows teachers shared their own computer interface to students in real time and builds a platform for teachers to share teaching resources and interact with students. Increased student focus is also achieved through the interactive software. The majority of the students agreed that the interactive software prevented them from using computers to do things that have nothing to do with learning during the PowerPoint presentation (76.93%). Throughout the whole teaching process, teachers could easily supervise the students by monitoring their computer interface and a black screen and text warnings were utilized to remind students who were not studying attentively.

Results of the questionnaire survey show that students are satisfied with the application of IVMLS and proves the above conjecture from the side. They supported that the application of IVMLS shortened the distance between them and teachers, and that it had the advantage of increased accessibility, high image quality, ease of use and perfect simulation of traditional microscope reading mode. They also felt that they were more willing to interact with classmates in IVMLS classes. In fact, what IVMLS brings is not limited to changes of technology and teaching materials, but the flipping of classroom mode. This new classroom model integrates various pedagogical methods, e.g., student-centred learning, collaborative learning and inquiry-based learning. It represents a model pedagogical tool that focuses on guided inquiry, understanding concepts, and turning from memory to critical thinking^24,46,58^.

We believe that IVMLS brings not only a direct change in student performance, but also a flip of classroom mode. The introduction of a new teaching tool encourages and facilitates teachers to adopt more advanced teaching strategies. In IVMLS classes, the teaching strategy of combining student-centred learning, inquiry-based learning and collaborative learning was adopted and believed to promote better understanding and mastery of knowledge^59,60^.

In this teaching mode, most of the time in the classroom is at the disposal of the students themselves. This type of learning is more likely to stimulate critical thinking, taking into account the individual level of students in order to develop their learning ability and investigative skills^61^. Developing student-centred interactive tools not only helps with the teaching of histopathology, but also helps students acquire group and self-monitoring skills^54^. In the process of independent exploration of slides, students were intentionally guided to think independently and avoid discussion, which forced them to actively learn, reason, understand theoretical knowledge and basic concepts, and combine static histological images with dynamic physiological functions. Student feedback also supports this process to promote their ability to think and reason independently. These independent activities are considered important for learning micromorphology^40,54,62^. During the free discussion in the second half of the class, students need to collaborate to deepen their understanding of theory and better prepare for homework. The use of virtual microscopes is thought to improve student collaboration as students can view exactly the same specimen, allowing for the same point of reference in group work and discussions^44,54,63,64^. More active classroom discussion and student collaboration were indeed observed in IVMLS group. Collaborative learning has its roots in the theories of social interdependence, cognitive development and behavioural learning and comes from the constructivism theory of social learning^54,65^. In constructivism theory, students play an active role in their own learning and construct new ideas on the basis of current and past knowledge. Collaborative learning not only has cognitive benefits, such as improved academic performance and motivation, but also improves the social skills needed for future professional work^65^.

### An inclusive environment

In traditional laboratories, the microscope devices between students were independent and the slides viewed were different. When they wanted to share images under their microscope with others, the other student needed to re-adjust the microscope to suit his viewing habits and needs. Similar things happened between students and teachers. In addition, when teachers wanted to share images under their microscopes with students, they could only line up and observe them one by one, which often led to overcrowding and inefficiency. The increased time cost led to a decrease in students' enthusiasm for interaction and classroom engagement, especially for those marginalized and unrepresentative students. Students who are shy or uncomfortable asking questions are often marginalized and may fail to achieve their full potential ^17,66^. In fact, we believe that IVMLS creates an inclusive environment which improves fairness and narrows the achievement gap between represented and underrepresented students ^21,67^. On the one hand, it avoids situations where classroom discussions are monopolized by overly active students, which can lead to marginalized students losing the opportunity to speak. Through decentring dominant groups, space can be made for marginalized voices and experiences^21^. On the other hand, the creation of a virtual platform for students to interact with teachers creates another option for students to interact with teachers, especially for those who are shy about face-to-face communication, thus creating a more inclusive environment where shy students obtained immediate feedback without the need to vocalize their questions^17^.

### Cumulative impact of IVMLS

Homework assignments required students to draw designated tissue structures after class to deepen their impression of the characteristics of various tissues. There was no significant difference in homework performance between the two groups of students. Students could discuss with each other when they were finishing their homework. They could also refer to any resources to complete the organization drawing task, such as pictures they downloaded in class or pictures in textbooks and on the Internet. The time to complete homework was not limited. Therefore, we think that students' homework performance can partly reflect students' learning attitude and their knowledge accumulated through in-class learning, but there are too many factors affecting students' homework performance, resulting in no significant difference in students' homework performance.

Significant differences were seen in laboratory test scores. We believe that the increase in learning resources and availability of virtual microscope platforms has increased the learning time students spend exploring lab courses. This is often long-term and sustainable process and often grows before the examination^40,43^. In fact, students who use VM spend more time watching virtual slides than students watch glass slides in the lab^68^. This indirectly proves that the duration of the use of virtual machine platform provides great advantages for microscope laboratory learning^43^, highlighting the cumulative impact of VM platform on laboratory performance^16,42,69^.

### An educational method suitable for generation Z students

Sociologists and researchers have studied the trends of five generations which were categorized as traditionalists, baby boomers, generation X, millennials and generation Z^70^. Generation Z includes those born between 1995 and 2012 and constitutes the majority of the current group of college students. They grew up under the influence of science and technology and never experienced life without the Internet. This makes generation Z students possess specific and unique characteristics compared with other generations. They usually have the technical skills required to participate in online learning and are eager for convenient, immediate and pragmatic learning^71^. But the limited experience of face-to-face communication may lead to their lack of social, interpersonal and communication skills^72^. Moreover, their limited attention may hinder the learning of online courses^73^.

In this context, IVMLS has advantages and disadvantages. First, generation Z students have the technical skills required to participate in online learning. Thus, they have the potential to rapidly adapt to IVMLS classes and fully explore and utilize this system. Secondly, IVMLS can be used to control students’ computers in the laboratory, monitor the tabs and programs displayed on their screen. This may help students concentrate and prevent them from using computers for activities that are unrelated to the class. The questionnaire results prove this view, showing that most students agreed that IVMLS prevented them from using computers to do things that have nothing to do with learning during the PowerPoint presentation. This makes up for the students' lack of concentration. In addition, students and teachers can choose to communicate online by sending messages to each other, which can make up for the shortcomings of students' lack of social and communication skills. This can reduce the obstacles of students' learning to a certain extent. But from another perspective, this cannot solve the plight of students' social skill inadequacies, but to a certain extent, it discourages their enthusiasm of face-to-face communication between students and teachers, making students more isolated.

### Potential application value of IVMLS

IVMLS can not only be used in laboratory sessions of oral histopathology, but also can be beneficial to other disciplines. It brings about the flip of classroom mode and represents a superior learning approach that appeals to more learning styles. Based on the system's powerful interaction characteristics and virtual microscope technology, various teaching methods, such as distance education, team-based learning, problem-based learning, cooperative education and peer education, have potential application space^52,69,74,75^.

IVMLS often comprises interactive software, teaching resource, slide-reading software and integrated auxiliary equipment. Some components of the system are also beneficial to other disciplines. The core part is the interactive software ParaSaga EClass. Through this software, teachers can share teaching resources, interact with students, share the computer interface with each student's computer in real time, and monitor the tabs and programs displayed on their screen. Students can also actively interact with teachers on this platform. Therefore, ParaSaga EClass interactive software can be applied to all kinds of computer related courses. But it is worth noting that the software requires 100 m and 1000 m Ethernet network environment, and that the switch is required to support and enable multicast protocol. Therefore, the software is mainly suitable for offline course teaching.

One of the integrated auxiliary equipment is a camera equipment installed on each console, which greatly expands the application of IVMLS, so that the system can also be applied to various operation courses. The camera device can record the teacher's operation and display it on the connected computer, and the image on the teacher's computer can be shared with students in real time. This greatly facilitates the development of operation courses, such as tooth preparation and root canal preparation, which are preclinical courses for stomatological students. In addition, IVMLS can help prevent cheating in exams, as students’ computer can be controlled by teachers’ host computer and students are effectively monitored by the camera equipment equipped on each console. Components of the IVMLS described here either exist in most biological laboratories or can be obtained at an affordable cost. The obvious impact of this technology on histological learning makes it cost-effective to invest in acquiring some of these components.

During COVID-19, some components of the system can be used for online courses. The virtual slide and slide-reading software brings great convenience to the transmission of teaching resources, so that students can break away from the limitations of the laboratory and study the laboratory course of histopathology online. Their combination with network conference software can effectively solve the problem of curriculum development. Although the effectiveness of this course form and its impact on students' learning effect need to be studied.

## Limitations

As IVMLS is based on the network, the efficiency of the class is greatly affected by the network condition. The shared interface may freeze, and some computers may lose control in the middle of the class due to the delay in the network. The disadvantages of virtual slides are also found in this teaching method. For instance, oil immersion lens cannot be applied to the scanning of classic oral histopathology slides by Nano Zoomer 2.0-HT system. Thus, higher magnification cannot be acquired at present. Additionally, although the images are compressed, they can still be extremely large with several gigabytes in size^76^. Hence, a limited selection of slides are provided as learning materials, and they may not be representative of the numerous inherent variations present, thus hindering the development of the practical and critical thinking abilities of the learners^30,77^.

It is worth noting that it is difficult to attribute the positive impact to any single component rather than the whole system. Research aimed at measuring the influence of various components of IVMLS, such as the slide-reading software and ParaSaga EClass would analyse the importance of these components.

## Conclusion

Our research results confirm the educational value of the proposed teaching method. IVMLS incorporates the advantages of interactive and virtual technology and creates a convenient interactive platform between teachers and students. Although certain limitations are present, solutions can be found during the study process. In general, IVMLS brings the flipping of classroom mode, integrates various pedagogical methods, and can promote students’ better understanding and mastery of knowledge.
